# P-2018. Nirmatrelvir versus Short-Course Remdesivir for Mild-to-Moderate COVID-19 in High Risk Hospitalized Patients: A Propensity Score-Matched Study

**DOI:** 10.1093/ofid/ofae631.2175

**Published:** 2025-01-29

**Authors:** Madeline Belk, Taylor Steuber, Jonathan Edwards

**Affiliations:** Huntsville Hospital, Huntsville, Alabama; UMKC School of Pharmacy, Columbia, Missouri; Huntsville Hospital, Huntsville, Alabama

## Abstract

**Background:**

As the Coronavirus disease 2019 (COVID-19) has evolved, patients often present with mild-to-moderate disease as opposed to severe disease that was seen earlier in the pandemic. Two previously published studies (PINETREE and EPIC-HR) evaluated treatment options in the outpatient setting for patients with mild-to-moderate disease. These studies may provide insight into how patients with similar disease presentation may be treated in the inpatient setting. The purpose of this study was to evaluate the use of nirmatrelvir (N/R) and short-course remdesivir in high-risk patients hospitalized with mild-to-moderate COVID-19.

Clinical Outcomes in the Propensity-Matched Cohort

**Figure:**
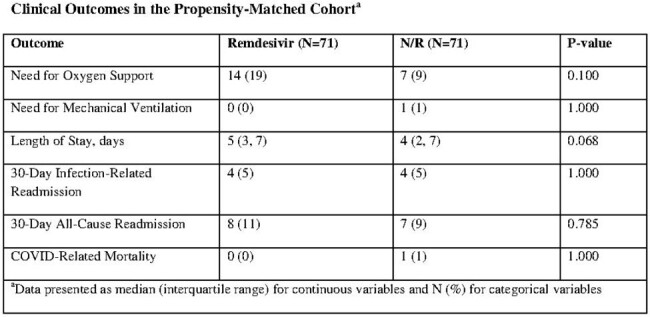

**Methods:**

This was a single-center, retrospective cohort study including adult patients hospitalized for mild-to-moderate COVID-19 between January 2021 and December 2023. Patients were grouped by treatment received (N/R or short-course [3-day] remdesivir). They were excluded if they received additional COVID-19 therapies, did not receive the appropriate regimen or were treated prior to hospitalization. Patients were compared by treatment received after propensity score-matching based on baseline characteristics. The primary endpoint assessed was the need for oxygen support. Exploratory endpoints included need for mechanical ventilation, length of stay, 30-day readmission, and mortality.

**Results:**

Eight-hundred and fifty-six patients were initially screened. After exclusions and propensity score-matching, a total of 75 matched pairs (N=150) were included in the analysis. Patients had an average of three comorbidities or high risk features for COVID-19 progression, most commonly age greater than 60 years, BMI over 25 kg/m^2^, hypertension, and diabetes. There was no difference in the primary outcome of need for oxygen support in the short-course remdesivir group compared to the N/R group (14 [19%] vs 7 [9%], respectively; p=0.100). There was also no difference in length of stay between the two groups (5 [3, 7] vs 4 [2, 7] days, respectively; p=0.068). All additional endpoints were similar between short-course remdesivir and N/R.

**Conclusion:**

Patients at high-risk of progression with mild-to-moderate COVID-19 that received N/R or short-course remdesivir had similar rates of disease progression, length of stay, readmissions, and mortality.

**Disclosures:**

Jonathan Edwards, PharmD, BCPS, BCGP, BCIDP, Gilead Sciences, Inc.: Advisor/Consultant|Gilead Sciences, Inc.: Honoraria

